# Endurance Exercise Enhances the Effect of Strength Training on Muscle Fiber Size and Protein Expression of Akt and mTOR

**DOI:** 10.1371/journal.pone.0149082

**Published:** 2016-02-17

**Authors:** Zuzanna Kazior, Sarah J. Willis, Marcus Moberg, William Apró, José A. L. Calbet, Hans-Christer Holmberg, Eva Blomstrand

**Affiliations:** 1 Swedish Winter Sports Research Centre, Department of Health Sciences, Mid Sweden University, Östersund, Sweden; 2 Swedish School of Sport and Health Sciences, Stockholm, Sweden; 3 Department of Physical Education, University of Las Palmas de Gran Canaria, Las Palmas de Gran Canaria, Spain; 4 Research Institute of Biomedical and Health Sciences (IUIBS), University of Las Palmas de Gran Canaria, Campus Universitario de Tafira s/n, Las Palmas de Gran Canaria, Canary Island, Spain; University of Birmingham, UNITED KINGDOM

## Abstract

Reports concerning the effect of endurance exercise on the anabolic response to strength training have been contradictory. This study re-investigated this issue, focusing on training effects on indicators of protein synthesis and degradation. Two groups of male subjects performed 7 weeks of resistance exercise alone (R; n = 7) or in combination with preceding endurance exercise, including both continuous and interval cycling (ER; n = 9). Muscle biopsies were taken before and after the training period. Similar increases in leg-press 1 repetition maximum (30%; P<0.05) were observed in both groups, whereas maximal oxygen uptake was elevated (8%; P<0.05) only in the ER group. The ER training enlarged the areas of both type I and type II fibers, whereas the R protocol increased only the type II fibers. The mean fiber area increased by 28% (P<0.05) in the ER group, whereas no significant increase was observed in the R group. Moreover, expression of Akt and mTOR protein was enhanced in the ER group, whereas only the level of mTOR was elevated following R training. Training-induced alterations in the levels of both Akt and mTOR protein were correlated to changes in type I fiber area (r = 0.55–0.61, P<0.05), as well as mean fiber area (r = 0.55–0.61, P<0.05), reflecting the important role played by these proteins in connection with muscle hypertrophy. Both training regimes reduced the level of MAFbx protein (P<0.05) and tended to elevate that of MuRF-1. The present findings indicate that the larger hypertrophy observed in the ER group is due more to pronounced stimulation of anabolic rather than inhibition of catabolic processes.

## Introduction

Concurrent training is commonly performed by both elite and recreational athletes. The early study by Hickson [1] indicated that endurance training may worsen adaptation to strength training when the two are performed in the same session. Subsequently, additional studies have provided support for negative interactions between endurance and strength training, attenuating the development of strength and power [2, 3]. However, not all studies have found negative effects of combined endurance and strength training [2, 3]. For example, Lundberg and colleagues [4, 5] reported recently that 5 weeks of concurrent training actually led to more pronounced increase in muscle volume, with the same increase in muscle strength. Thus, some uncertainty remains concerning the influence of endurance exercise on the hypertrophic response to resistance exercise.

Activation of the Akt-mTOR (mechanistic target of rapamycin) pathway is considered to make a major contribution to the development of muscle mass and, indeed, this pathway is activated by different types of exercise [6]. In rodent muscle, activation of AMP-activated protein kinase (AMPK) suppresses protein synthesis and attenuates signaling through the mTOR pathway [7], observations that have led to the proposal of a mechanism for interaction between endurance and strength training: the elevation of AMPK activity induced by endurance exercise inhibits mTOR signaling during a subsequent session of resistance exercise. This hypothesis has been tested in humans employing various combinations of acute endurance and resistance exercise [8–12]. Although the findings to date do not provide support for AMPK-mediated inhibition of mTOR signaling during the recovery period after exercise, the effect of long-term concurrent training on adaptation of the Akt-mTOR pathway has not yet been investigated.

The effect of concurrent training on muscle strength has been relatively well documented, but less is known about the long-term effects of such training on muscle growth, fiber composition, capillary density, and oxidative capacity. Resistance exercise is known to stimulate anabolic processes, leading to enlargement of fiber areas [13, 14]. At the same time, eight weeks of strength training was found to stimulate expression, both at the mRNA and protein levels, of the ubiquitin ligases MAFbx and MuRF-1, suggesting activation also of processes regulating protein breakdown in muscle [15]. However, whether the strength training-induced enlargement in fiber area and the elevations of markers for protein synthesis and degradation are affected by prior endurance exercise is not known.

In the present investigation, two groups of subjects trained for seven weeks, one combining endurance and strength training in the same sessions and the other performing strength training only. The study was designed to further examine whether endurance training, with session of high-intensity intervals, influences the response to a subsequent session of resistance exercise with regard to strength development, muscle fiber composition, and fiber size, as well as total level of proteins in the Akt-mTOR pathway. In addition, expression of MAFbx and MuRF-1 at both the mRNA and protein levels was evaluated.

## Materials and Methods

### Subjects

Sixteen healthy men, who did not perform endurance or resistance exercise on a regular basis participated in the study. After being fully informed about the purpose of the study and associated risks they provided their written informed consent to participate. These subjects were divided into two groups with similar physical characteristics (see Table 1). The study protocol was approved by the Regional Ethical Review Board in Umeå, Sweden, and performed in accordance with the principles outlined in the Declaration of Helsinki.

**Table 1:** Physical characteristics of the two groups of subjects.

Group	Age (years)	Height (cm)	Body mass (kg)	1RM (kg)	$\dot{\text{V}}$O_2max_ (l/min)
R (n = 7)	28 ± 3.7	182 ± 6.0	77 ± 6.7	292 ± 25.7	3.68 ± 0.64
ER (n = 9)	26 ± 5.3	179 ± 9.6	78 ± 12.1	282 ± 27.7	3.51 ± 0.90

The values presented are means ± SD for the number of subjects given in parenthesis. R: resistance training group, ER: combined endurance and resistance training group.

### Determination of submaximal and maximal oxygen uptake

All testing sessions were carried out on a cycle ergometer (Schoberer Rad Messtechnik, Julich, Germany). Each subject adjusted the seat height and handlebar position for his own comfort and these settings were noted and employed in subsequent sessions of testing. Oxygen uptake ($\dot{\text{V}}$O_2_) was determined at 3–4 submaximal work rates, starting at 80 W with subsequent increases of 30 W. Following a 10-min recovery, a maximal incremental test was performed, starting at 80 W with 30 W increases every minute until exhaustion. Oxygen uptake was monitored continuously by an on-line system (AMIS 2001 model C; Innovision A/S, Odense, Denmark) and the average values during the final 30 s at each work rate were calculated. Heart rate (HR) was monitored continuously using the Polar S610 apparatus (Polar Electro OY, Kempele, Finland). Work rates corresponding to approximately 65% and 90% of $\dot{\text{V}}$O_2max_ were calculated from these measurements.

### Measurements of one-repetition maximum (1RM)

The 1RM was determined on a leg press machine (Free Motion EPIC PlateLoaded Leg Press, F218, Utah, USA) as previously described by Baechle and Earle [16]. In short, after warming up (15 repetitions at 40% of predicted 1RM, followed by 6 repetitions at 60–70% of predicted 1RM), the load was gradually increased until the participant was unable to perform more than one single repetition (from a 90° to 180° knee angle)**.** Initially 30–40 kg was added to the last warm-up load, then the load was increased (5 to 20 kg) on the basis of the subject’s performance at the previous level. Subjects were given 3–5 attempts to perform their 1RM with 3 minutes between each trial.

All testing (submaximal and maximal oxygen uptake and 1RM) was performed at least 5 days before the start of the training period and at least 48 hours after completion of the training period. The two types of tests were always performed on different days and, as far as possible at the same time of day pre- and post-training for each subject.

### The training program

The resistance training group (R) performed leg press exercise only, whereas the concurrent training group performed endurance exercise (cycling) followed by 10 min rest and then resistance exercise (ER). The subjects were instructed to continue their regular activities of daily living (i.e. walking and cycling), without participating in any other training program.

The training period lasted for seven weeks, with two sessions in each of the first two weeks and progressively increased to four sessions in weeks six and seven, giving a total of 21 sessions (see Table 2 for more detailed description). Leg press exercise started at a load corresponding to 70% of the individual’s initial 1 RM and this load was raised 5–7% every third or fourth training session, to match the observed gain in strength. The number of sets was increased from four at week one to six at week five and number of repetitions in each set decreased from twelve to eight with a 3-min rest between sets. The subjects were guided to perform each repetition at a set pace, i.e., with concentric and eccentric phases of 2 s each. This resistance exercise protocol lasted for about 30 min and care was taken to maintain the same intensity for both groups.

**Table 2:** The resistance training program.

Week	Session	Sets	Repetitions
1–2	1–4	4	12
3–4	5–10	5	10
5–6	11–17	6	8
7	18	6	8
	19	4	15, 12, 8, until failure
	20	4	15, 12, 8, until failure
	21	4	15, 12, 8, until failure

Endurance sessions on a cycle ergometer (Monark 828E, Monark Exercise AB, Vansbro, Sweden) were performed at a work rate corresponding to 63 ± 1.2% of $\dot{\text{V}}$O_2max_ with the training intensity being increased progressively every two weeks to maintain a constant relative work rate. The subjects were instructed to keep a steady self-paced cadence, which turned out to be 60–65 rpm. Sessions of interval cycling at a work rate corresponding to 95 ± 1.8% of $\dot{\text{V}}$O_2max_ were incorporated into the final three weeks (sessions no 12, 14, 16, 18, 20) to enhance activation of AMPK [17, 18]. For further details of the training progression, see Table 3. All training sessions by both groups were carefully supervised.

**Table 3:** The endurance training program for the ER group.

Session	Duration	Intensity
1	30 min	60% of $\dot{\text{V}}$O_2max_
2–3	40 min	60% of $\dot{\text{V}}$O_2max_
4–6	50 min	60% of $\dot{\text{V}}$O_2max_
7–10	55 min	60% of $\dot{\text{V}}$O_2max_
11, 13, 15, 17, 19	60 min	60% of $\dot{\text{V}}$O_2max_
12	51 min	6 x 2 min at 95% of $\dot{\text{V}}$O_2max_
14, 16	57 min	7 x 2 min at 95% of $\dot{\text{V}}$O_2max_
18, 20	63 min	8 x 2 min at 95% of $\dot{\text{V}}$O_2max_
21	30 min, taper	60% of $\dot{\text{V}}$O_2max_

The number of sessions each week was the same as in the resistance training program, with endurance training being carried out prior to resistance training. All endurance sessions also included a 5-min warm-up and a 5-min cool-down.

Within 20 min after completion of a training session, subjects in the R-group received a protein supplement (Kolozzeum Pure Whey, Stockholm, Sweden), 20 g dissolved in 500 ml of water to enhance muscle recovery. The ER-group were given this same supplement, but with addition of maltodextrin (Fairing Fast Carbs, Järfälla, Sweden) in an amount corresponding to the individual´s calculated energy expenditure during the endurance training.

### Muscle biopsies

Biopsies were taken from the lateral part of m. quadriceps, i.e., the *vastus lateralis*, both before and after 7 weeks of training. The subjects were instructed to refrain from training for 2 days prior to the pre-training biopsies and the post-training biopsies were taken approximately 2 to 3 days after the final session in 15 subjects, but in one subject the post-training biopsy was taken 90 hours after the final session. During this period the subjects also refrained from training.

The subjects arrived at the laboratory in the morning following an overnight fast, with the same procedure pre- and post-training. After 10 min of supine rest, the skin above the middle portion of the *vastus lateralis* was anesthetized with 2% lidocaine (B. Braun Medical, Danderyd, Sweden) and biopsies taken using the needle technique with suction [19, 20]. The pre-training biopsies were taken from the right leg of 8 subjects and the left leg of the other 8. To reduce methodological error two biopsies for histochemical analyses were taken from most of the subjects [21]. The post-training biopsies were taken 2–3 cm proximal (8 subjects) or distal (8 subjects) to the pre-training one. The tissue obtained was rapidly cleaned from blood and fat and divided into two parts, one part was immediately frozen in liquid nitrogen. The other part was mounted in an embedding medium (Tissue Tek^®^ O.C.T. Compound) and frozen in isopentane cooled to its freezing point in liquid nitrogen for histochemical analyses. The samples were then stored at -80°C until being analyzed.

#### Histochemical analysis

Serial 10-μm cross-sections were cut in a cryostat at -20°C. Following preincubation at pH 4.3, 4.6 and 10.3, the sections were stained for myofibrillar ATPase at pH 9.4 [22]. The muscle fibers were classified as type I, IIA, IIB or IIC [23], although we use the new terminology of types I, IIA and IIX, since human skeletal muscle does not contain the fast-type IIB fibers present in small animals [24]. To visualize capillaries, the cross-sections were stained by the amylase-PAS procedure [25].

Computer image analysis (Leica QWin Runner V 3.5.1, Leica Microsystems, Bromma, Sweden) was performed to evaluate capillaries, fiber composition and fiber areas.

At least 150–200 fibers were classified and analyzed at each time point and areas were determined for a minimum of 15 fibers of each type as recommended previously [21, 26]. The average number of fibers analyzed in each biopsy sample was for type I fibers 146 (range 26–419), for type IIA fibers 75 (range 23–152) and for type IIX fibers 59 (range 18–176). Only in 7 subjects (3 in the ER-group and 4 in the R group), the number of IIX fibers exceeded 15 in both pre- and post-biopsies.

#### Enzyme assays

For measurements of enzyme activities, the muscle tissue that was frozen immediately in liquid nitrogen was utilized. These samples were lyophilized and blood, fat, and connective tissue removed. Approximately 3 mg was then homogenized in ice-cooled extraction medium (50 mM Tris HCl, 5 mM MgCl_2_, 1 mM EDTA, pH 8.2) with a ground-glass homogenizer (100 μl per mg dry muscle). The maximal activities of citrate synthase [27] and 3-hydroxyacyl CoA dehydrogenase [28] were assayed under optimal conditions as described previously. Measurements were performed in a Beckman Coulter DU 800 spectrophotometer (Beckman Coulter Inc., Brea, CA) at 25°C. Analyses were performed in 13 subjects (8 in the ER and 5 in the R group) from whom sufficient muscle tissue was available.

#### Quantification of mRNA

Total RNA was extracted from 2–3 mg lyophilized muscle tissue by homogenization in PureZOL RNA Isolation Reagent (Bio-Rad Laboratories, Sundbyberg, Sweden) using a Polytron (Kinematica, Luzern, Switzerland). The concentration and purity of the isolated RNA were assessed spectrophotometrically as described previously [29]. One microgram of this RNA was then utilized to generate 20 μl of complementary DNA (cDNA) with the iScript cDNA Synthesis Kit (Bio-Rad Laboratories). The concentration of cDNA, annealing temperature, and conditions for the polymerase chain reaction (PCR) were optimized for each primer pair and maintained within the linear range of amplification. To allow direct comparison of pre- and post-samples from each participant, the samples were run in triplicate in parallel on the same 96-well plate.

Real-time quantitative PCR (RT-qPCR) was performed on a Bio-Rad iCycler (Bio-Rad Laboratories) in a 25-μl solution containing 12.5 μl SYBR Green Supermix (Bio-Rad Laboratories), 0.5 μl each of the forward and reverse primers (10 μmol/l) and 11.5 μl template cDNA (for further details, see [29]). The housekeeping GAPDH mRNA was used as an internal control, which has previously been validated under the same experimental conditions [29, 30]. Expression of each target gene was evaluated with the 2^-ΔCt^ procedure, where ΔC_t_ represents the difference between the Ct for the gene of interest and that of GAPDH [31].

#### Immunoblotting

Approximately 3 mg lyophilized muscle was homogenized in ice-cold buffer (80 μl/mg) containing 20 mM HEPES, pH 7.4, 1 mM EDTA, 5 mM EGTA, 10 mM MgCl_2_, 50 mM β-glycerophosphate, 1 mM Na_3_VO_4_, 2 mM DTT, 1% Triton X-100, 20 μg/ml leupeptin, 50 μg/ml aprotinin, 40 μg/ml PMSF and 1% phosphatase inhibitor cocktail (Sigma P-2850). These homogenates were then centrifuged at 10,000 g for 10 min at 4°C to remove cell debris and the supernatant collected and its protein concentration determined by the BCA protein assay (Pierce Biotechnology, Rockford, IL). Aliquots of the supernatant (diluted to a protein concentration of 3.0 μg/μl with homogenizing buffer) were mixed with an equal volume of Laemmli buffer and heated for 5 min at 95°C to denature proteins. Then 30 μg protein was loaded onto an SDS-PAGE gel and run on ice at 200 V for 120 min to separate proteins. Both samples from the same subject were run on the same gel.

The gel was then incubated in transfer buffer (25 mM Tris-HCl, 192 mM glycine, 20% methanol) at 4°C for 30 min before electrophoretic transfer of the proteins to a polyvinylidine fluoride membrane (Bio-Rad, Laboratories, Richmond, CA) for 3 h on ice in a 4°C cold room. The membrane was subsequently blocked at room temperature for 1 h in TBS (Tris buffered saline; 10 mM Tris pH 7.6, 100 mM NaCl) containing 5% non-fat dry milk and then incubated overnight at 4°C with commercially available primary antibodies diluted in TBS containing 0.1% Tween 20 (TBST) with 2.5% non-fat dry milk: GAPDH (1:5,000), Akt (1:1,000), mTOR (1:1,000), S6K1 (1:1,000) purchased from Cell Signaling Technology (Beverly, MA); α-tubulin (1:5,000) from Sigma-Aldrich (St. Louis, MO); MAFbx (1:1,000) from Abcam (Cambridge, UK) and MuRF1 (1:1,000) from Santa Cruz Biotechnology (Santa Cruz, CA). The latter two antibodies have recently been validated using MAFbx and MuRF-1 transfected HEK293 cells and a negative non-transfected control [32].

The membranes were then washed in TBST and incubated with a secondary antibody (anti-rabbit, anti-mouse (1:1,000) purchased from Cell Signaling Technology (Beverly, MA) or anti-goat (1:1,000) from Abcam (Cambridge, UK)) for 1 h at room temperature prior to serial washing and visualization of the proteins by enhanced chemiluminescence using the Molecular Imager ChemiDoc XRS system (Bio-Rad Laboratories). All bands were analyzed using the box tool in the Quantity One version 4.6.3 software (Bio-Rad Laboratories) and protein levels expressed as arbitrary units relative to GAPDH, since this protein was unaffected by the training protocols. In contrast, the level of α-tubulin, which is often used as a reference protein, was increased after both training protocols.

### Statistical analysis

All data are expressed as means ± SD and were checked for normal distribution before performing parametric statistical analyses. A two-way repeated measures ANOVA (time, group) was applied to evaluate and compare the effect of training in the R and ER groups. When the ANOVA showed a significant main effect or interaction between time and group, Fisher’s LSD post hoc test was applied to identify where the differences occurred. A P-value <0.05 was considered to be statistically significant.

## Results

### Muscle strength and oxygen uptake

The different training regimes resulted in the same enhancement in muscle strength (main effect of time; P<0.01). 1RM increased by 30%, from 292 ± 25.7 to 378 ± 37.4 kg in the R group and from 282 ± 27.7 to 367 ± 31.2 kg in the ER group. The progressive increase in relative work load during the seven-week training period was also similar in the two groups (data not shown). Body weight increased slightly but significantly in both groups (main effect of time; P<0.05), from 76.6 ± 6.68 to 78.6 ± 8.16 kg in the R group and from 77.5 ± 12.1 to 78.7 ± 12.0 kg in the ER group.

The maximal $\dot{\text{V}}$O_2_ increased from 3.51 ± 0.90 to 3.80 ± 0.78 l/min (P<0.05) in the ER group, but remained unchanged in the R group (3.68 ± 0.64 pre- vs. 3.69 ± 0.56 l/min post-training). Also when expressed per kg body weight, the maximal oxygen uptake increased from 45.1 ± 8.5 to 48.2 ± 6.9 ml/min/kg (P<0.05) in ER group, but remained unchanged in the R group (48.3 ± 8.3 pre- vs. 47.3 ± 7.9 ml/min/kg post-training). In addition, time to exhaustion during the maximal test was improved from 8.52 ± 2.24 to 9.43 ± 2.19 min (P<0.05) in the ER group, but was unaltered in the R-group (9.07 ± 1.51 pre- vs. 8.97 ± 1.55 min post-training). For all three variables, the ANOVA revealed a significant interaction (time and group), in addition a significant main effect of time was detected for VO_2max_ (l/min) and time to exhaustion.

### Enzyme activities

In the ER-group, the maximal activity of citrate synthase (CS) was 21% higher (58.2 ± 12.1 to 70.2 ± 12.2 μmol/min/g, P<0.05) after the training period, whereas the maximal activity of HAD was unchanged (39.8 ± 11.0 and 40.9 ± 6.7 μmol/min/g before and after training, respectively). The corresponding values for CS in the R group were: 56.3 ± 6.6 and 58.6 ± 4.8 μmol/min/g and for HAD: 45.1 ± 5.6 and 41.6 ± 4.2 μmol/min/g before and after training, respectively. For CS activity, the ANOVA revealed a main effect of time as well as a significant interaction (time and group).

### Histochemistry

Seven weeks of ER training reduced the relative proportion of type I-fibers from 56 to 46% (P<0.05; main effect of time and interaction time and group), without significantly influencing the proportion of type IIA and type IIX fibers (Table 4). The R training did not significantly alter the fiber composition, although in both groups the proportion of type IIC (intermediate) fibers tended to increase by training (P = 0.072; main effect of time) (Table 4).

**Table 4:** Fiber composition, fiber area, and capillary density in biopsies from the vastus lateralis taken before (Pre) and after (Post) 7 weeks of training.

	Condition			
	R Pre	R Post	ER Pre	ER Post
Type I (%)	58 ± 11.4	57 ± 11.5	56 ± 16.4	46 ± 16.1*
Type IIA (%)	27 ± 8.8	29 ± 10.1	33 ± 11.4	38 ± 9.2
Type IIX (%)	15 ± 9.8	13 ± 10.9	11 ± 13.4	13 ± 11.5
Type IIC (%)	0.3 ± 0.4	0.9 ± 0.6	0.9 ± 1.1	2.2 ± 2.3
Type I area (μm^2^)	4900 ± 435	4750 ± 708	4500 ± 1380	5610 ± 1550*
Type IIA area (μm^2^)	5300 ± 875	5670 ± 614*	5680 ± 962	7130 ± 2140*
Type IIX area (μm^2^)	4300 ± 288	4740 ± 444*^1^	4800 ± 1521	5530 ± 1623*^2^
Mean fiber area (μm^2^)	4910 ± 445	4970 ± 546	4840 ± 1250	6210 ± 1830*
Capillaries/fiber	1.86 ± 0.16	1.97 ± 0.16*	1.94 ± 0.50	2.19 ± 0.49*
Capillaries/mm^2^	350 ± 45.6	358 ± 46.6	358 ± 35.5	338 ± 40.0

R: resistance training and ER: combined endurance and resistance training. The ANOVA revealed a main effect of time and interaction (time and group) for the proportion of type I fibers, mean fiber area and type I fiber area. A main effect was revealed for the area of type IIA and IIX fibers as well as capillaries per fiber. The values presented are means ± SD for 7 subjects in the R group and 9 subjects in the ER group, unless otherwise indicated.

*P<0.05 for Post vs. Pre training

^1^ n = 4

^2^ n = 3

The mean fiber area increased significantly only in the ER-group, 28% vs. virtually no increase in the R-group (Table 4). The ER protocol increased the areas of type I, IIA and IIX fibers by 25, 26 and 15%, respectively, whereas only the type IIA and IIX fiber areas were significantly enhanced, 7% and 10%, respectively in the R-group (Table 4). The ANOVA revealed a main effect of time (P<0.01) as well as interaction time and group (P<0.01) for the mean fiber area and type I area as well as a main effect of time P<0.05) for the areas of type IIA and IIX.

The number of capillaries per fiber was elevated by 5% and 13% after 7 weeks training in the R and ER group, respectively (main effect of time; P<0.05). The number of capillaries per square mm was not altered in any of the groups (Table 4).

### Levels of mRNA and total protein

**Fig 1:**
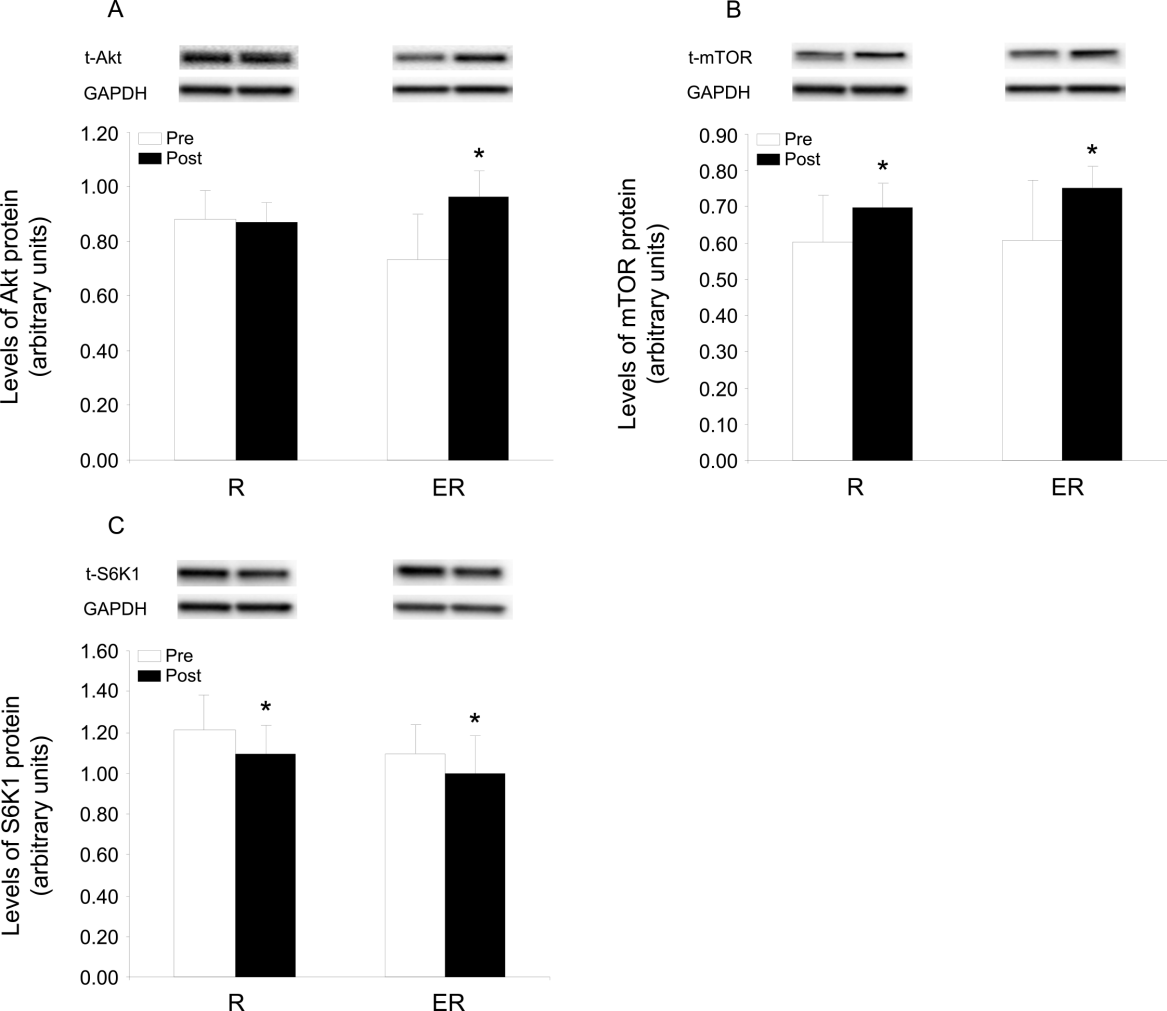
Levels of proteins in the Akt signaling pathway before and after 7 weeks of training. (A) Akt, (B) mTOR and (C) S6K1 in skeletal muscle before (Pre) and after (Post) 7 weeks of strength training only (R) or combined endurance and resistance exercise (ER). The values presented are arbitrary units normalized to the corresponding level of GAPDH and represent means ± SD for 7 subjects in the R group and 9 subjects in the ER group. For all three proteins, the ANOVA revealed a main effect of time, in addition a significant interaction (time and group) was detected for levels of Akt. Representative immunoblots from two subjects (one from each group) are shown above of each graph. *P < 0.05 for Post vs. Pre training.

The level of Akt protein was 32% higher after the ER training, but unchanged in the R group (Fig 1A; main effect of time (P<0.01) and interaction time and group (P<0.01)). The content of mTOR protein was increased 24% and 15% in ER and R, respectively, while that of S6K1 fell by approximately 10% in both groups (Fig 1B and 1C), with the ANOVA revealing a main effect of time (P<0.05) in the case of both these proteins. Neither regime significantly influenced the levels of mRNA coding for proteins considered to regulate mTOR activity (Rheb, REDD1 and 2, data not shown).

**Fig 2:**
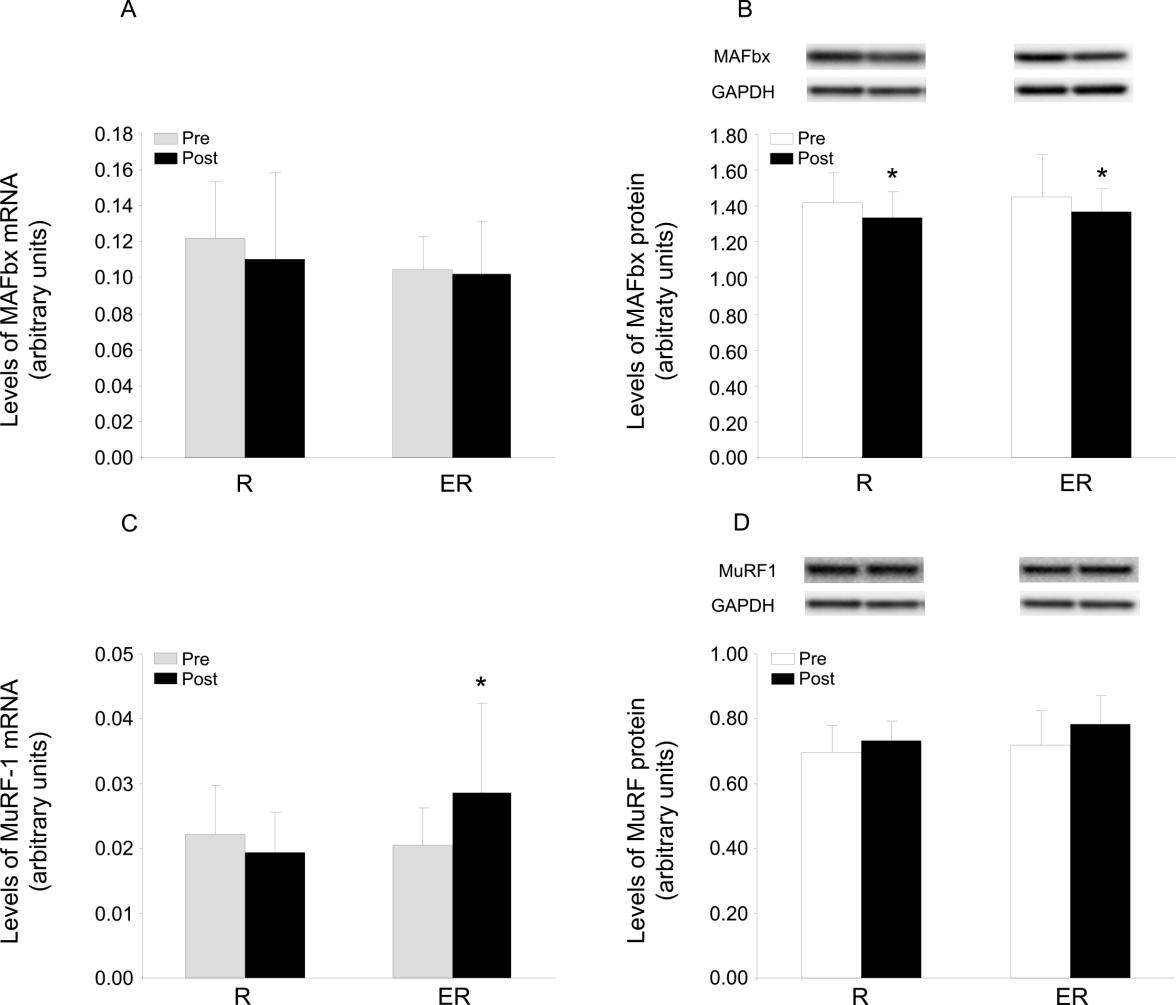
Expression of MAFbx and MuRF-1 before and after 7 weeks of training. (A) mRNA and (B) protein levels of MAFbx, (C) mRNA and (D) protein levels of MuRF-1 in skeletal muscle before (Pre) and after (Post) 7 weeks of strength training only (R) or combined endurance and resistance exercise (ER). The values presented are arbitrary units normalized to the corresponding level for GAPDH and represent means ± SD for 7 subjects in the R group and 9 subjects in the ER group. The ANOVA revealed a main effect of time for total protein levels of MAFbx as well as an interaction (time and group) for mRNA expression of MuRF-1. Representative immunoblots from two subjects (one from each group) are shown above graphs C and D. *P < 0.05 for Post vs. Pre training.

Expression of the ubiquitin ligases was to some extent influenced differently by the two training regimes. The level of MuRF-1 mRNA was elevated in the ER, but not the R group (an interaction time and group was revealed in the ANOVA). In contrast, the level of MuRF-1 protein tended to be higher (P = 0.082; main effect of time) following both ER and R training. The level of MAFbx mRNA was not altered in either case, although the level of the corresponding protein was reduced by both forms of training (Fig 2; a significant main effect of time).

### Correlations

**Fig 3:**
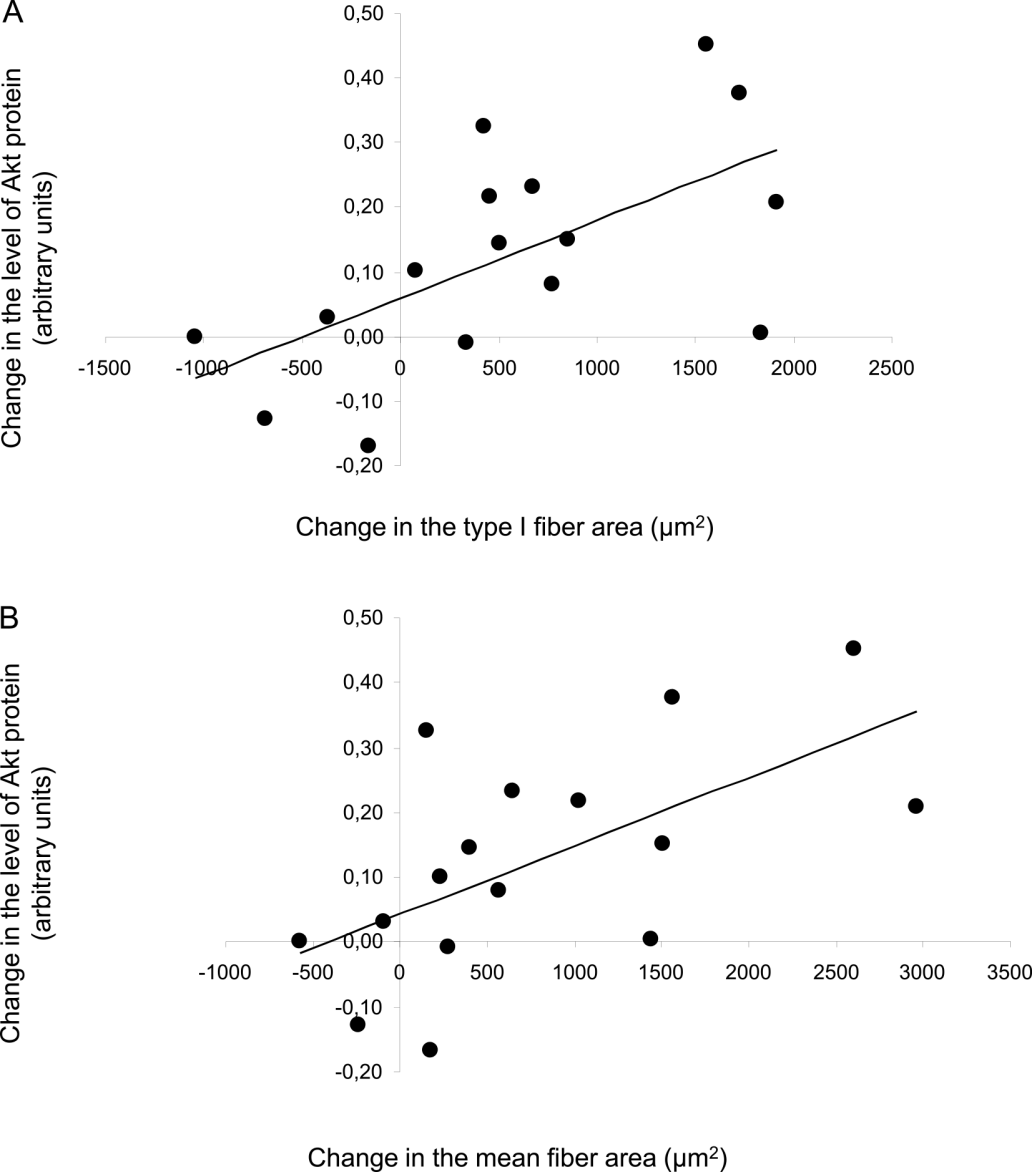
Relationship between changes in Akt protein and changes in fiber area. (A) correlation between changes in the level of Akt protein and changes in type I fiber area (r = 0.61; P<0.05), (B) correlation between changes in the level of Akt protein and changes in mean fiber area (r = 0.61; P<0.05).

**Fig 4:**
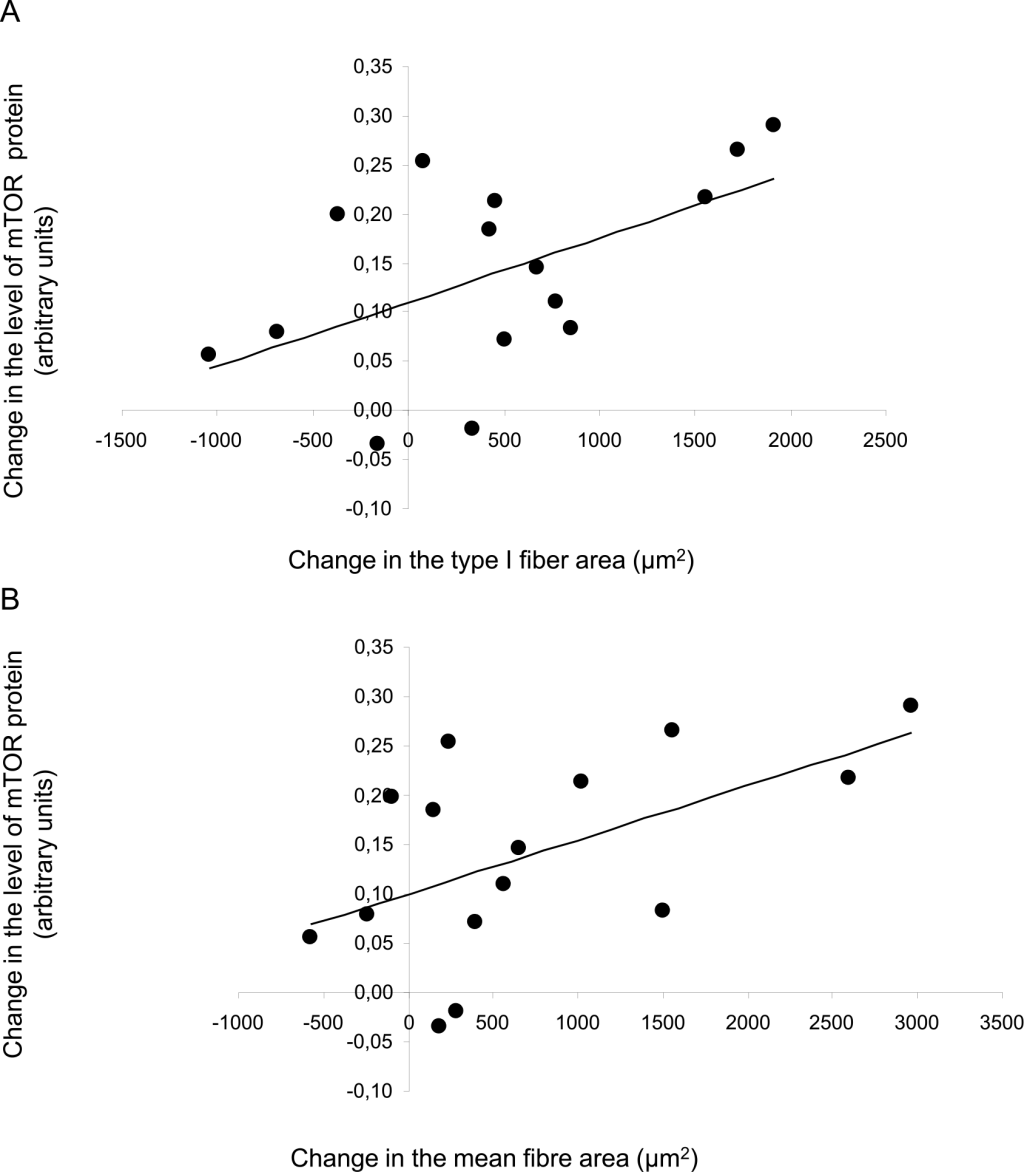
Relationship between changes in mTOR protein and changes in fiber area. (A) correlation between changes in the level of mTOR protein and changes in type I fiber area (r = 0.55; P<0.05), (B) correlation between changes in the level of mTOR protein and changes in mean fiber area (r = 0.55; P<0.05). One value differed considerably from the rest and was therefore regarded as an outlier and excluded from the calculations (1830; -0.16 and 1438; -0.16 in A and B, respectively).

There was a significant correlation between the changes in the level of Akt protein and the changes in both type I fiber area (r = 0.61, P<0.05) and the mean fiber area (r = 0.61, P<0.05; Fig 3A and 3B), but not with changes in the type IIA or IIX fibers. Also changes in mTOR protein correlated significantly with changes in type I fiber area (r = 0.55, P<0.05) and mean fiber area (r = 0.55, P<0.05, Fig 4A and 4B), but not with changes in the type IIA or IIX fibers.

## Discussion

The major finding in the present study was that endurance training does not impair the enhancement in maximal strength or fiber hypertrophy induced by a subsequent session of strength training. Actually, the combined training led to more pronounced increase in muscle fiber area, accompanied by elevations in the levels of both Akt and mTOR proteins in the *vastus lateralis* muscle. In addition, the combined training enhanced the area of both type I and type II fibers, whereas strength training only increased the type II fibers.

However, the larger increase in muscle fiber area and thus putative enlargement in muscle volume following combined endurance and strength training were not reflected in more extensive improvement of muscle strength. The 1 RM was elevated to the same extent by both training protocols, in agreement with a recent report by Lundberg and colleagues [5], where the subjects performed unilateral training for 5 weeks. This suggests that the improvement in maximal strength (1RM) observed following our relatively short 7-week period of training is due largely to neuromuscular adaptation.

Seven weeks of combined training enhanced $\dot{\text{V}}$O_2max_ and the maximal activity of the TCA cycle enzyme citrate synthase, in agreement with previous findings on endurance training [33]. Unexpectedly, the maximal activity of HAD, which has been reported to change in a manner similar to the TCA cycle enzymes [28, 34], was unchanged. Thus, it is possible that the strength training may have counteracted the expected increase in HAD activity after endurance training. In addition, the number of capillaries per fiber increased, although the number of capillaries per square mm was unaltered as a result of the pronounced muscle fiber hypertrophy.

In agreement with previous reports, our strength training protocol did not induce changes in $\dot{\text{V}}$O_2max_, citrate synthase or HAD activity [35, 36]. However, Tang and coworkers [14] did observe elevated oxidative capacity (CS and HAD activities) following 12 weeks of strength training. The reason for the divergent results in these studies is not clear but the use of different training protocols and differently trained subjects may partially explain the various results.

The concurrent training protocol reduced the proportion of type I fibers and tended to increase the proportion of type IIA fibers, whereas strength training had no such effects. A reduction in the proportion of type I fibers following combined training has not been reported previously, but an increase in type IIA fibers is commonly found, often accompanied by a corresponding reduction in the proportion of type IIX fibers [13]. Reduction in the proportion of type I fibers, as well as a rise in the proportion of type IIA fibers have in fact been observed following 4–6 weeks of sprint training or 9 weeks of strength training combined with plyometric jumps [37, 38]. In these studies, as well as in our combined group, a transition from type I to type IIA fibers appears to reflect a functional adaptation, although the underlying mechanisms remain unclear.

Although both training regimes resulted in hypertrophy, they influenced the two main fiber types differently. Concurrent training enlarged the areas of both type I and type II fibers, whereas strength training only increased the type II fiber areas. This suggests an additive effect of endurance and strength training on muscle hypertrophy as well as a qualitatively different response. Earlier reports that endurance training increases both type I and type II fibers in previously untrained subjects [39], support our assumption of an additive effect of the two types of training, although it cannot be ruled out that the effect is due to the greater amount of work carried out by the ER group. Furthermore, the relatively large effect in this group can probably be attributed to the untrained nature of the subjects since the acute activation of intracellular signaling to both endurance and resistance exercise is more pronounced in untrained than trained muscle [40, 41]. With respect to strength training alone, our observations agree with some other studies, although increases in both main fiber types have been found by others [13, 14]. Different training regimes, including the intensity, volume, and duration of the training period, probably explain, at least in part, the various findings.

The two training regimes induced different changes in proteins involved in the regulation of anabolic processes in muscle with the combined training protocol leading to enhanced levels of both Akt and mTOR protein, whereas strength training only increased mTOR protein content (Fig 1). Acute endurance exercise is known to stimulate Akt signaling, whereas resistance exercise stimulates mTOR activity, often, but not always without the involvement of Akt [30, 42, 43]. Furthermore, eight weeks of strength training was reported to increase mRNA expression of mTOR and raptor, a protein that binds to mTOR and is essential for mTOR complex 1 activity [44]. This observation suggests that in addition to acute activation of mTOR, strength training also modulates the gene expression of mTOR. Assuming that the training effect is due to the cumulative effects of repeated sessions of training, the present findings might have been predicted; however, increases in the levels of Akt and mTOR protein in human muscle have not been demonstrated previously.

To our knowledge, only in one previous study on humans has the effect of strength training on the levels of anabolic proteins been studied, and somewhat different results were presented [45]. Twelve weeks of one-leg concentric or eccentric resistance exercise had no effect on the level of Akt or mTOR protein despite a 6–8% increase in quadriceps cross-sectional area [45]. There is no obvious explanation to the partly divergent results, although the different types of exercise employed might contribute to the diverse response.

The divergent influence of our two training protocols, i.e., enhanced levels of both Akt and mTOR protein in the combined group but only increased content of mTOR in the R group, might be the mechanism underlying the more pronounced increase in mean fiber area caused by combined training. Alternatively, it could be argued that activation of Akt-signaling during endurance exercise relates to its role in glucose metabolism [46, 47] rather than in anabolic signaling. Speaking against this view is the observation that the level of Akt protein was similar in endurance trained and sedentary individuals [48]. Moreover, the correlations observed between alterations in the expression of these proteins and both the mean fiber area and type I fiber area (Figs 3 and 4) provide further support for their involvement in muscle hypertrophy, as previously demonstrated for rodent muscle [49]. Based on the present findings it is tempting to suggest that activation of both Akt and mTOR potentiates stimulation of protein synthesis.

In contrast to the enhanced levels of Akt and mTOR protein, both training regimes led to a reduction in the level of S6K1, a protein downstream of mTOR that is often used as an indicator of mTORC1 activation. Although, the reason for the divergent regulation of these proteins is unclear, it is possible that up-regulation of S6K1 may amplify its inhibitory feedback on the insulin receptor substrate (IRS) 1, which impairs the activation of phosphoinositide 3-kinase (PI3K) and Akt [50, 51]. In addition, S6K1 might be expressed at high levels in human *vastus lateralis* muscle, so that a certain extent of down-regulation therefore does not attenuate its ability to stimulate protein synthesis. However, at present, it is difficult to speculate on the significance of the reduction in the level of S6K1. Further studies to explore the effects of training on the capacity of the mTOR-signaling pathway are required.

The subjects in the present study received a protein supplement following completion of each training session in order to stimulate protein synthesis and standardize their dietary intake during the early recovery period. Recently, Camera and colleagues [52] reported that anabolic processes were stimulated more potently by concurrent exercise (leg extension followed by 30 min of cycling) when a protein beverage was consumed immediately afterwards. A similar effect has also been observed following resistance exercise [53]. Therefore, it is unlikely that the supplements we employed elicited different responses in the two groups. The subjects performing concurrent exercise were also given carbohydrates to compensate for their energy consumption during cycling, but this is unlikely to have influenced the anabolic response since it has been shown that carbohydrates have little or no stimulatory effect on muscle protein synthesis [54].

A training-induced increase in muscle volume requires that the rate of protein synthesis exceeds that of protein breakdown, which over time will lead to accretion of muscle mass. In skeletal muscle, the proteasome system involving the ubiquitin ligases MAFbx and MuRF-1, is considered to play a major role in the degradation processes [55, 56]. Furthermore, activation of the protein Akt has been linked not only to anabolic processes but also to catabolic reactions by inhibiting the transcriptional up-regulation of ubiquitin ligases [57–59]. Therefore, it might be expected that these processes would be attenuated in the ER group. However, both training regimens induced similar changes in protein levels of the two ubiquitin ligases, i.e. they tended to elevate the level of MuRF-1 protein while lowering the level of MAFbx protein. These observations indicate that the alterations in mRNA levels following acute exercise observed previously in a number of studies [29, 30, 60–63] are in fact reflected in changes in protein levels. This, in turn, provides further support for the proposal that these two ubiquitin ligases play different roles in connection with protein breakdown, muscle remodeling and adaptation to training [29, 30]. However, the predicted inhibitory effect of Akt-activation on the ubiquitin ligases in the ER group did not occur, based on the similar changes in both training regimes. Accordingly, our present findings indicate that the more pronounced fiber hypertrophy in the ER group is due more to stimulation of anabolic than inhibition of catabolic processes.

In summary, the current investigation provides additional evidence that endurance exercise does not compromise the anabolic stimulus provided by subsequent strength training. Our combined training enhanced the expression of both Akt and mTOR protein, as well as the areas of type I and type II fibers, whereas strength training only led to elevated protein content of mTOR and increased size of type II fibers. In addition, correlations between changes in the levels of Akt and mTOR protein and changes in type I area and the mean fiber area indicate that these proteins play an important role in hypertrophy. We also found dissociation between mTOR upregulation and its downstream target S6K1, which may have implications for the sensitivity in this pathway, however this remains to be determined. The combined training reduced the proportion of type I fibers, but no change in fiber composition was observed following strength training alone.
